# Schema-driven facilitation of new hierarchy learning in the transitive inference paradigm

**DOI:** 10.1101/lm.030296.113

**Published:** 2013-07

**Authors:** Dharshan Kumaran

**Affiliations:** Institute of Cognitive Neuroscience, University College London, London WC1N 3AR, United Kingdom

## Abstract

Prior knowledge, in the form of a mental schema or framework, is viewed to facilitate the learning of new information in a range of experimental and everyday scenarios. Despite rising interest in the cognitive and neural mechanisms underlying schema-driven facilitation of new learning, few paradigms have been developed to examine this issue in humans. Here we develop a multiphase experimental scenario aimed at characterizing schema-based effects in the context of a paradigm that has been very widely used across species, the transitive inference task. We show that an associative schema, comprised of prior knowledge of the rank positions of familiar items in the hierarchy, has a marked effect on transitivity performance and the development of relational knowledge of the hierarchy that cannot be accounted for by more general changes in task strategy. Further, we show that participants are capable of deploying prior knowledge to successful effect under surprising conditions (i.e., when corrective feedback is totally absent), but only when the associative schema is robust. Finally, our results provide insights into the cognitive mechanisms underlying such schema-driven effects, and suggest that new hierarchy learning in the transitive inference task can occur through a contextual transfer mechanism that exploits the structure of associative experiences.

Prior knowledge, in the form of a mental schema or framework of associative information, is thought to facilitate the learning of new information in a range of experimental and everyday scenarios (Bartlett 1932; Brandsford 1979; McClelland et al. 1995; Maguire et al. 1999; Tse et al. 2007; Kumaran et al. 2009; van Kesteren et al. 2010a,b, 2012; Tse et al. 2011). In recent years, there has been increasing interest in specific neural mechanisms that underlie this process (Tse et al. 2007; Kumaran et al. 2009; van Kesteren et al. 2010a,b, 2012; Tse et al. 2011). For example, an influential study in rodents found that the learning of new associations (i.e., a new flavor–place paired associate) was markedly enhanced in the presence of an associative schema consisting of previously acquired flavor–place associations, experienced in a similar context (i.e., the same event arena) (Tse et al. 2007). Strikingly, this speeded (i.e., one-trial) associative learning became rapidly hippocampal-independent, providing evidence for a schema-driven shift in the neural mechanisms underlying the learning of new information.

Despite the surge of interest in the cognitive and neural mechanisms underlying the facilitatory effects of an associative schema on new learning, there is a paucity of experimental paradigms specifically designed to address this question in humans. In this study, we examine schema-based effects in the context of the transitive inference (TI) paradigm—an experimental scenario which provides a well-controlled setting in which to examine the influence of an associative schema (i.e., prior knowledge of the rank position of items in the hierarchy) on the learning of new information (i.e., relating to the rank position of novel items). To the best of our knowledge, however, the TI paradigm has not been employed with this purpose in mind, though it has been widely used across species to study the mechanisms that support inferential behavior and generalization, and in particular the specific contribution of the hippocampus (Bryant and Trabasso 1971; McGonigle and Chalmers 1977, 1992; Harris and McGonigle 1994; Rapp et al. 1996; Dusek and Eichenbaum 1997; Delius and Siemann 1998; Greene et al. 2001; Frank et al. 2003, 2005; Heckers et al. 2004; Titone et al. 2004; Smith and Squire 2005; Moses and Ryan 2006; Moses et al. 2010; Kumaran et al. 2012; Zeithamova et al. 2012).

In this three-phase study involving 30 participants, we used a version of the TI paradigm, which follows the lines of the original paradigm developed by Bryant and Trabasso (1971), and has been shown to ensure the development of robust transitivity performance across a group of participants, underpinned by relational knowledge of the hierarchy (see Materials and Methods) (Cohen and Eichenbaum 1993; Eichenbaum 2004; Smith and Squire 2005; Kumaran et al. 2012). Our experimental design (Fig. 1; Materials and Methods) allowed us to ask whether prior knowledge about the position of items in a previously learned seven-item hierarchy (i.e., associative schema acquired in phase 1) would facilitate learning of the position of novel items in a new nine-item hierarchy (i.e., during phase 2). Critically, the use of a control condition during phase 2, which involved learning of an entirely novel nine-item hierarchy, enabled us to identify the presence of specific schema-driven learning effects, uncontaminated by general changes in strategy. Finally, in phase 3, we probed the underlying mechanism of schema-driven facilitatory effects—specifically, a contextual transfer mechanism whereby rank information spreads from familiar items in the hierarchy (i.e., the schema) to novel items simply through their association (e.g., from A to B during an AB training trial)—by asking whether participants could learn new information even in the absence of corrective feedback. Furthermore, we manipulated the strength of the associative schema (i.e., between subject groups; see Materials and Methods and below), and asked whether successful performance through contextual transfer would only be possible in the “strong associative schema” group (i.e., Group II).

**Figure 1.**
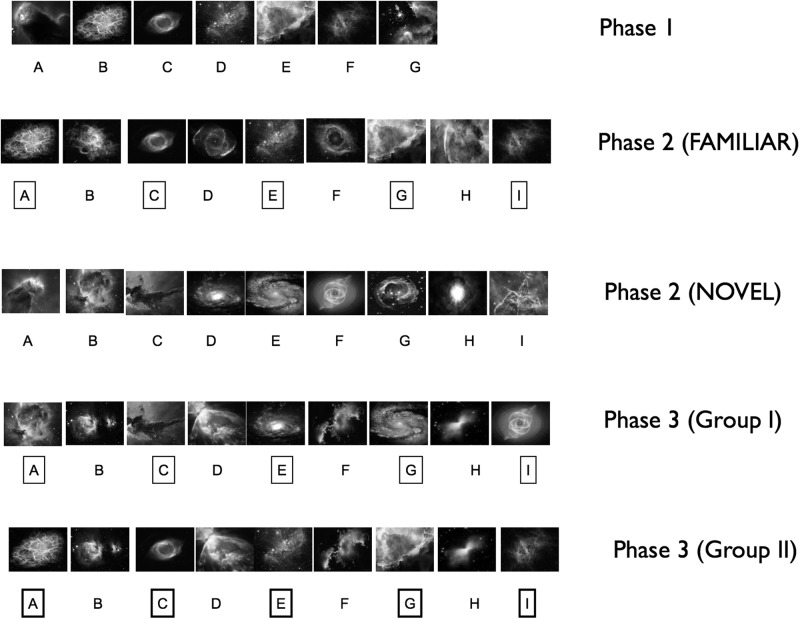
Composition of hierarchies used in phases 1–3. Familiar items surrounded by square boxes indicate those that have been seen before in a previous phase. Novel items are shown unboxed. Very familiar items (i.e., items in the Group II phase 3 hierarchy seen in both phases 1 and 2) are shown enclosed in bold square boxes. Specifically, the phase 2 FAMILIAR hierarchy comprised five items (i.e., A, C, E, G, I) that had been previously part of the phase 1 hierarchy (i.e., in the B–F positions). In phase 3, hierarchy composition varied according to participant group: In Group I, five of the items (i.e., A, C, E, G, I) in the phase 3 hierarchy were derived from the NEW hierarchy in phase 2 (i.e., the B, C, D, E, F items, respectively). In Group II, five of the items (i.e., A, C, E, G, I) were derived from the FAMILIAR hierarchy in phase 2 (i.e., the A, C, E, G, I items, respectively). For both groups, the other four items (i.e., B, D, F, H) were novel and had not been previously experienced (see Materials and Methods for details). Note that the letter labels (e.g., “A”) denote ordinal positions, and are shown purely for explanatory purposes—these labels were never shown to participants. Note also that items are depicted in grayscale, but were presented in color.

## Results

During all three experimental phases, participants completed blocks of training trials, which were followed by blocks of test trials. Training trials involved the presentation of adjacent items (i.e., galaxies) in the hierarchy (e.g., phase 1: six premise pairs A vs. B, B vs. C, C vs. D, D vs. E, E vs. F, F vs. G); participants were required to select the item which they thought was “older,” with corrective feedback issued in phases 1 and 2 (though not phase 3). Test trials also required participants to choose the galaxy which they thought was “older,” but differed from training trials in two principal ways: First, nonadjacent items in the hierarchy were presented (e.g., phase 1: B–D, B–E, B–F, C–E, C–F, D–F). Second, corrective feedback was not presented during test trials. As such, participants were required to use a capacity for transitive inference to deduce the correct item during test trials.

### Phase 1

**Figure 2.**
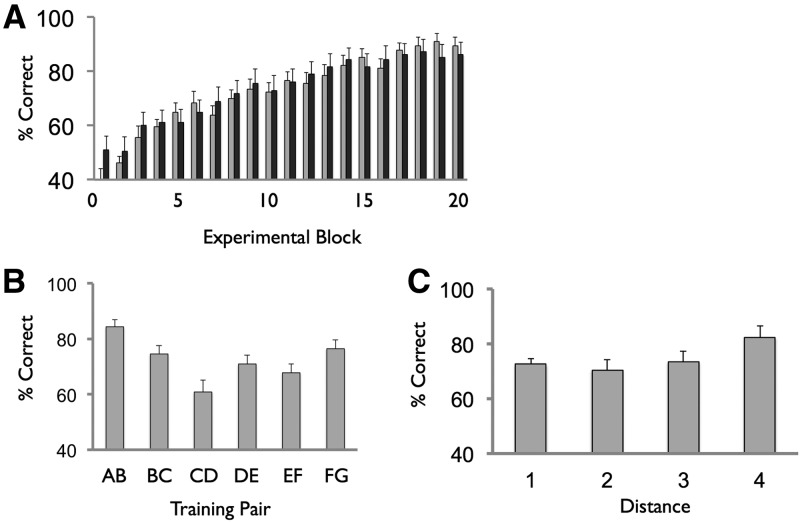
Phase 1 performance. (*A*) Training trial (light gray) and test trial (dark gray) performance shown across the 20 blocks. (*B*) Training trial performance as a function of pair. (*C*) Test trial performance as a function of symbolic distance (e.g., a B–D test trial has a distance of two). Note that test trial blocks also included training pairs (e.g., AB), denoted by a distance of one—these are shown for completeness here, but not included in the statistical analysis of the symbolic distance effect (see Results). Data averaged across all 30 participants. Error bars reflect SEM.

By the end of this phase, participants reached high levels of performance on both training (mean 89.5%, SEM 3.0) and test (mean 86.0%, SEM 4.7) trials (Fig. 2A). Data were analyzed using an ANOVA with within-subject factors of block (20 levels), trial type (two levels: train, test). Though both participant groups were treated equivalently in this phase, a between-subjects factor of group was also included. In terms of percentage of correct responses, we observed a significant main effect of block (*F*_(6.9,194.4)_ = 29.2, *P* < 0.001), indexing the improvement of training and test trial performance over the experimental session (Fig. 2A). No significant effects were observed in relation to any of the other factors or interactions between factors (all *P*’s > 0.1).

We next considered the performance of participants on training trials as a function of training pair. Data were collapsed across all training trial blocks and analyzed using a mixed-design ANOVA with one within-subject factor of training pair type (six levels: A vs. B, B vs. C, …, G vs. H), and one between-subject factor of group. For % correct responses, we observed a significant main effect of training pair type (*F*_(5,140)_ = 8.3, *P* < 0.001), with a significant quadratic effect (*F*_(1,28)_ = 28.9, *P* < 0.001) (Fig. 2B). For RT we also observed a main effect of training pair type (*F*_(1.2,32.7)_ = 3.4, *P* < 0.01), with a significant quadratic effect (*F*_(1,28)_ = 21.5, *P* < 0.001). No effect of group or group by training pair interaction was observed (*P* > 0.1). These results, therefore, are consistent with the operation of associative learning mechanisms during training trials (Moses and Ryan 2006; Zeithamova et al. 2012).

We next examined how performance on test pairs varied as a function of the distance between the position of items in the hierarchy (i.e., symbolic distance, e.g., equal to two for a B vs. D trial, and four for a B vs. F trial) (Fig. 2C). Relating to percentage of correct responses, an ANOVA with factors distance (two, three, four) and group showed a significant main effect of distance, *F*_(1.5,41.2)_ = 10.9, *P* < 0.001, characterized by a linear effect, *F*_(1,28)_ = 15.4, *P* < 0.001. No other significant effects were observed (*P* > 0.1). We found a similar pattern in terms of RT: main effect of distance, *F*_(1.6,44.8)_ = 9.7, *P* < 0.001, characterized by a linear effect, *F*_(1,28)_ = 10.3, *P* < 0.001. Though we did not ask participants to explicitly recall the underlying hierarchy at the end of phase 1—so as to avoid biasing participants’ strategy during phase 2—the profile of findings observed, in conjunction with those of previous studies (e.g., Kumaran et al. 2012), suggests that by the end of this experimental phase participants had acquired robust relational knowledge of the underlying hierarchy (see Discussion).

### Phase 2

In this phase, participants were exposed to two nine-item hierarchies: One hierarchy, termed “NEW,” was comprised entirely of novel items that had never before been experienced (Fig. 1; Materials and Methods). The other hierarchy, termed “FAMILIAR,” was comprised of five items that had been part of the phase 1 hierarchy, and four novel items that had not been seen before. Critically, the relative order of the five familiar items was preserved between the phase 1 and FAMILIAR hierarchies, such that they effectively formed a pre-established scaffold (i.e., A, C, E, G, I), in between which the novel items (i.e., B, D, F, H) were inserted (see Materials and Methods for details). Further, this method of constructing the FAMILIAR hierarchy (i.e., inserting novel items between familiar items) ensured that new learning was required to determine the correct choice for each training pair—and also that all test pairs involved choices between items that were novel for phase 2. Note that both participants groups were treated identically during this phase, with the exception that those in Group I were exposed to the FAMILIAR hierarchy in the first train–test cycle, whereas those in Group II were exposed to the NEW hierarchy (i.e., to control for possible order effects). Further, the allocation of individual stimuli was counterbalanced such that the items making up the FAMILIAR hierarchy for one subject group were used in the NEW hierarchy for the other group (and vice versa).

**Figure 3.**
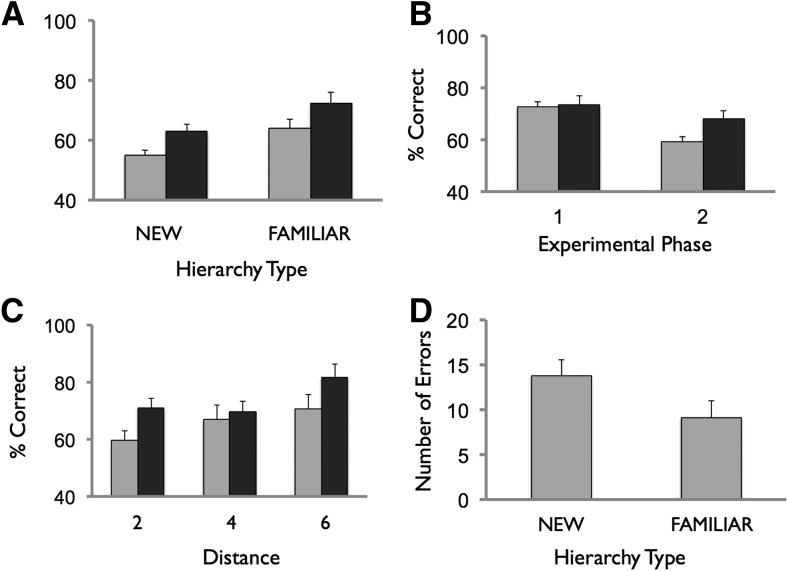
Phase 2 performance. (*A*) Training trial (light gray) and test trial (dark gray) performance shown for NEW and FAMILIAR conditions (i.e., performance averaged across all blocks in phase). (*B*) Training trial (light gray) and test trial (dark gray) performance shown for phases 1 and 2 (i.e., performance averaged across all blocks in a phase). (*C*) Test trial performance as a function of symbolic distance, NEW (light gray), FAMILIAR (dark gray). (*D*) Performance on hierarchy recall test in NEW and FAMILIAR conditions. See Materials and Methods for scoring scheme. Note, chance performance has mean 26 errors (SD 6) based on simulation (involving 1000 randomly generated hierarchies). Data averaged across all 30 participants. Error bars reflect SEM.

The experimental design of phase 2, therefore, was configured to examine participants’ ability to use a prior schema (i.e., the rank positions of familiar items in the phase 1 hierarchy) to facilitate new learning in the FAMILIAR condition, as compared to the NEW comparison condition. Performance averaged across all blocks for the FAMILIAR hierarchy for training and test trials was 63.0% (SEM 2.2) and 72.4% (SEM 3.7), respectively (Fig. 3A). For the NEW hierarchy, training trial performance was 55.2% (SEM 1.8) and test trial performance 64.0% (SEM 3.1). Data were analyzed using a mixed-design ANOVA with within-subject factors of hierarchy type (FAMILIAR, NEW), block (12 levels), and trial type (two levels: train, test) with one between-subjects factor of group also included. In terms of % correct responses, we observed a significant main effect of trial type (*F*_(1,28)_ = 14.5, *P* < 0.001), and of block (*F*_(3.5,96.8)_ = 27.4, *P* < 0.001), with a linear effect (*F*_(1,28)_ = 84.0, *P* < 0.001). Critically, there was a significant main effect of hierarchy type (*F*_(1,28)_ = 10.6, *P* < 0.01). As such, participants performed significantly better on both training and test trials in the FAMILIAR condition, as compared to the NEW condition. No other effects, including the effect of group and interactions, were significant (*P*’s > 0.1). The superior performance in the FAMILIAR, as compared to the NEW, condition was confirmed in subsequent ANOVAs that examined training and test trials separately. As such, there was a significant main effect of hierarchy type in relation to training trials (*P* < 0.01), and test trials (*P* < 0.05).

It is interesting to note that test trial performance (i.e., averaged across phase 2) was significantly higher than training trial performance, in both FAMILIAR and NEW conditions (Fig. 3A), evidenced by the significant main effect of trial type in the ANOVA presented above (*P* < 0.001). This contrasts with a general trend in the literature for training trial performance to be numerically superior or comparable to test trial performance (e.g., Moses and Ryan 2006), as well as the pattern of findings observed in phase 1. Specifically, in phase 1, training and test trial performances (i.e., averaged across the phase) were comparable (mean training trial, 72.5% [SEM 2.0]; test trial, 70.6% [SEM 3.6]; no significant difference, *P* > 0.1) (Fig. 3B). Indeed, when performance was directly compared between phases 1 and 2 (i.e., an ANOVA with factors phase (1, 2) and trial type [training, test]), there was a significant interaction between phase and trial type (*F*_(1,28)_ = 8.2, *P* < 0.01) that reflected the superiority of test trial performance (cf. training) in phase 2 but not phase 1. Importantly, this finding cannot be accounted for by a difference in the average symbolic distance relating to test trials in phases 1 and 2—this parameter was equated by only including test trials with a symbolic distance of two (e.g., B vs. D) in both phases. Although it is important to bear in mind that phases 1 and 2 involved hierarchies of differing lengths (i.e., seven vs. nine) and different amounts of training (e.g., 12 vs. 20 blocks), the significant superiority of test trial performance in phase 2 could potentially reflect a change in learning strategy. One possibility is that having completed phase 1 participants were biased toward searching for an underlying hierarchical structure in phase 2, consistent with the notion of an “overhypothesis” that can constrain the learning of new information (e.g., Kemp et al. 2007).

We next examined how performance on test pairs during phase 2 varied as a function of the distance between the position of items in the hierarchy (e.g., B vs. H = symbolic distance of six) (Fig. 3C). Relating to % correct responses, an ANOVA with factors hierarchy type (FAMILIAR, NEW), distance (two, four, six), and group showed a significant main effects of hierarchy type (*F*_(1,28)_ = 5.1, *P* < 0.05), and distance (*F*_(2,56)_ = 12.7, *P* < 0.001). The effect of distance was characterized by a linear effect (*F*_(1,28)_ = 25.4, *P* < 0.001). No other significant effects were observed (*P* > 0.1).

Participants’ ability to accurately recall the order of items in the two hierarchies was tested at the end of phase 2 (see Materials and Methods). Nine/30 participants were able to perfectly recall the FAMILIAR hierarchy, compared to 4/30 participants in the NEW hierarchy. The average number of recall errors made was 9.1 (SEM 1.8) and 13.8 (SEM 1.9) for the FAMILIAR and NEW hierarchies, respectively (Fig. 3D). Data were analyzed in a mixed-design ANOVA with factors hierarchy type (FAMILIAR, NEW) and group. There was a significant main effect of hierarchy type (*F*_(1,28)_ = 4.5, *P* < 0.05), but no effect of group or interaction (*P* > 0.1), affirming the superior hierarchy recall accuracy relating to performance in the FAMILIAR, as compared to NEW, conditions.

The findings from phase 2 provide evidence that the presence of an associative schema in the FAMILIAR condition has a specific effect on facilitating performance in the TI paradigm, and ultimately results in more robust relational knowledge of the hierarchy.

### Phase 3

In phase 3, we examined whether an associative schema could support successful transfer under “extreme” conditions—we removed the provision of corrective feedback from training trials (see Materials and Methods), such that no feedback of any kind was presented during this phase. As such, successful (i.e., above chance performance) on training and test trials—as well as the hierarchy recall test that followed the end of the experimental phase—was only possible by generalizing (or transferring) the rank of familiar items in the hierarchy to novel items that had not been experienced before. Furthermore, we manipulated the strength of the associative schema (i.e., a between-group manipulation; see Materials and Methods and below), and asked whether successful performance would only be possible in the “strong associative schema” group (i.e., Group II).

In this phase, participants were exposed to one hierarchy, which consisted of five items that had been part of a hierarchy learned during phase 2 (Group I, items derived from phase 2 NEW hierarchy; Group II, items derived from the FAMILIAR hierarchy), and four items that had never before been experienced. In both subject groups the relative order of the previously learned items was preserved between the phase 2 and phase 3 hierarchies. Note that the procedure for constructing the hierarchy during phase 3 followed a similar method as that used during phase 2 (i.e., novel items were inserted between familiar items). As previously, therefore, this ensured that all training pairs involved a novel and a familiar item—and also that all test pairs involved choices between items that were novel for phase 3.

Critically, prior hierarchy knowledge could be considered to be stronger in Group II, as compared to Group I, for three reasons (see Materials and Methods): First, in Group II, the previously experienced items had not only been part of the phase 2 (FAMILIAR) hierarchy but also of the phase 1 hierarchy. Second, in Group II, the previously experienced items occupied exactly the same rank positions (i.e., A, C, E, F, H) as they had in the previous phase (i.e., in the FAMILIAR hierarchy). Finally, hierarchy recall accuracy was found to be significantly greater for the FAMILIAR hierarchy, as compared to the NEW hierarchy, at the end of phase 2.

**Figure 4.**
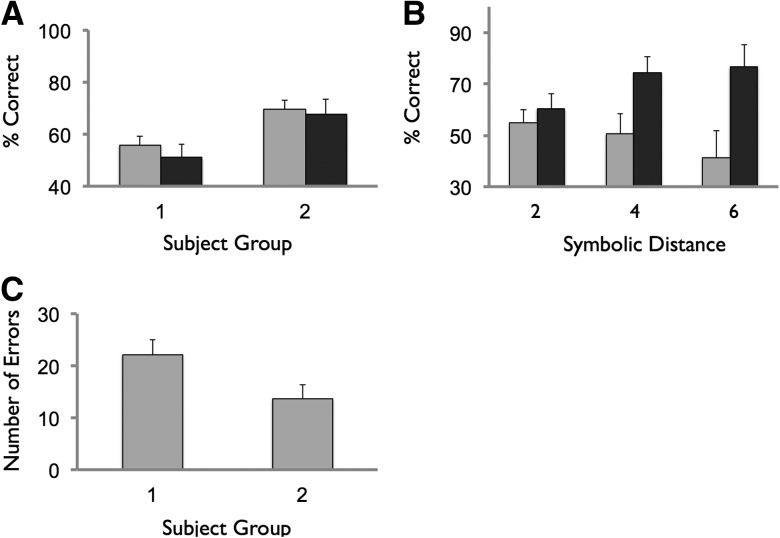
Phase 3 performance. (*A*) Training trial (light gray) and test trial (dark gray) performance shown for subject Groups I and II (i.e., performance averaged across all blocks in phase). (*B*) Test trial performance as a function of symbolic distance, Group I (light gray), Group II (dark gray). (*C*) Performance of each subject group on hierarchy recall test. See Materials and Methods for scoring scheme. Note chance performance has mean 26 errors (SD 6), based on simulation (involving 1000 randomly generated hierarchies). Error bars reflect SEM.

Average performance across the phase on training and test trials was Group I 55.8% (SEM 3.4) and 51.1% (SEM 4.9), respectively, and Group II 69.6% (SEM 3.6) and 67.8% (SEM 5.8) , respectively (Fig. 4A). A one-way ANOVA with factors group showed a significant main effect of group (*F*_(1,28)_ = 8.5, *P* < 0.01), confirming the superior performance of participants in Group II (i.e., the strong schema condition). Indeed, the performance of Group I did not rise significantly above chance performance during any of the six training or testing blocks (one-sample *t*-test, *P* > 0.1). Further, the average training and test trial performance during phase 3 of Group II showed a robust correlation with the number of errors made when asked to recall the FAMILIAR hierarchy at the end of phase 2 (i.e., training trial, *r* = −0.65, *P* < 0.01; test trial, *r* = −0.77, *P* < 0.001).

We next examined how performance on test pairs during phase 3 varied as a function of the distance between the positions of items in the hierarchy (Fig. 4B). An ANOVA with factors distance (two, four, six) and group showed the expected main effect of group (*F*_(1,28)_ = 7.5, *P* < 0.01). Further, there was a significant group × distance interaction (*F*_(2,27)_ = 27.0, *P* < 0.01), that was characterized by a linear effect (*P* < 0.01), reflecting that the superior performance of the strong schema group (cf. weak schema group) became more apparent with increasing symbolic distance.

Participants’ ability to accurately recall the order of items in the two hierarchies was tested at the end of phase 3 (see Materials and Methods). One/15 participants in Group I were able to perfectly recall the hierarchy, compared to 3/15 participants in Group II. The average number of recall errors made was 22.1 (SEM 2.9) and 13.7 (SEM 2.6) for Groups I and II, respectively (Fig. 4C). Notably, the hierarchy recall performance of Group II (*t*_(14)_ = −4.9 *P* < 0.001), but not of Group I (*P* > 0.1), was found to be significantly above chance performance (i.e., mean number of errors 26 [SD 6], generated using a simulation of 1000 randomly generated hierarchies).

Data were analyzed in a one-way ANOVA with factor group. There was a significant main effect of group (*F*_(1,28)_ = 6.1 *P* < 0.05), confirming the higher accuracy of hierarchy recall in the strong schema group (i.e., Group II), as compared to the weak schema group (i.e., Group I). Indeed, this effect remained significant when the hierarchy recall analysis was restricted solely to considering the relative order of items that were novel to phase 3 (i.e., the B, D, F, H items in the hierarchy).

## Discussion

Despite recent interest in the cognitive and neural mechanisms underlying schema-driven facilitation of new learning, few paradigms have been developed to examine this issue in humans. Our study provides a new paradigm to study these effects using a well-established experimental scenario, the transitive inference task. We demonstrate that an associate schema has a marked and specific effect on the learning of a new hierarchy that cannot be accounted for by less-specific changes in general strategy. Further, we show that participants are capable of deploying prior knowledge to successful effect under surprising conditions (i.e., when feedback is entirely absent), but only when the associative schema is robust. Finally, our results provide insights into the cognitive mechanisms underlying such schema-driven effects, and suggest that new hierarchy learning can occur through a contextual transfer mechanism that exploits the structure of associative experiences.

Although we intentionally did not require participants to explicitly recall the underlying hierarchy at the end of phase 1 (to avoid influencing participants’ strategy during phase 2) the pattern of data observed suggests that by the end of phase 1 participants had acquired robust relational knowledge of the underlying hierarchy. Although associative learning mechanisms are known to produce a linear symbolic distance effect, the high levels of transitivity performance achieved in the context of a relatively long (i.e., seven-item) hierarchy strongly favors an underlying relational hierarchy representation (Frank et al. 2003; Moses and Ryan 2006; Kumaran et al. 2012; Zeithamova et al. 2012). Further, this conclusion is also consistent with the results of other studies that have used an analogous version of the TI paradigm (i.e., Kumaran et al. 2012), where similar patterns of behavioral performance were demonstrated in association with highly accurate recall of the underlying hierarchy.

Our findings from phase 2 provide evidence that the presence of an associative schema in the FAMILIAR condition has a specific effect on facilitating performance in the TI paradigm, and ultimately results in more robust relational knowledge of the hierarchy. Importantly, in both FAMILIAR and NEW condition, new learning was required to support successful performance during training and test trials; further, test trials in both conditions were restricted to the novel items. Furthermore, through the use of the NEW baseline condition we were able to show that this effect cannot be explained by less-specific effects such as a change in overall learning strategy, as could potentially have been the case if we had set out to elicit schema-driven effects through comparing performance across different phases (e.g., phases 1 and 2). Further, the superior performance in the FAMILIAR as compared to the NEW condition cannot be a reflection of order effects, or mere stimulus-specific differences: Order and stimulus allocation was counterbalanced between subject groups (see Materials and Methods).

Future studies are required to examine the neural mechanisms underlying the schema-driven facilitation of new learning in the FAMILIAR condition. However, a recent study points toward a hippocampally mediated effect, by providing evidence that this brain region supports the acquisition of linear hierarchies in a domain-general fashion and generates a neural signal that linearly codes for rank information (Kumaran et al. 2012). As such, it is worth bearing in mind that the neural representation of associative schemas—and therefore the neural mechanisms of schema-driven facilitatory effects on new learning—are likely to differ depending on the experimental scenario under consideration. For instance, the study by Tse et al. (2007) suggests that neocortical-based schemas arise following extensive training (e.g., weeks) on flavor–place associations in an environment through systems-level consolidation, contrasting markedly with the hippocampal-dependence of a schema-driven enhancement of new learning that was based on a set of associative rules that had been recently and rapidly acquired (Kumaran et al. 2009).

The findings from phase 3 demonstrate that successful transitivity, training trial, performance, was possible, even in the absence of any corrective feedback, but only under conditions where prior knowledge was robust (i.e., strong schema, Group II). Further, this was associated with the development of relational knowledge of the underlying hierarchy, as measured by the hierarchy recall test, which was found to be present even when only novel items were considered. These results provide insights into the cognitive mechanisms underpinning such schema-driven enhancements of transitivity performance and relational hierarchy knowledge. Specifically, our findings support the hypothesis that prior knowledge concerning the rank positions of the familiar (i.e., A, C, E, G, I) items facilitates learning of the rank position of the novel items (i.e., B, D, F, H) through a form of “contextual transfer” (cf. Howard et al. 2005; Wimmer and Shohamy 2012). As such, participants might learn that the B item is lower in rank than A, but higher than C, through the spread of rank information during training trials (e.g., from the familiar A item to the novel B item, during an AB trial). According to this mechanism, new learning occurs simply by *viewing* the B item in association with the A and C items during (i.e., A–B, B–C) training trials, rather than through the provision of corrective feedback during trial-and-error learning (i.e., A+B). Notably, however, learning the rank position of novel items through contextual transfer must necessarily involve knowledge, be it “implicit” or “explicit,” of the structure of training trial presentation (i.e., that B appears only with A and C, C with B and D, etc.). If the schedule of training trial presentation was different—for example involving each item (e.g., A) appearing with all others (i.e., B–I)—then contextual transfer of this nature would not be a viable mechanism.

Interestingly, though multiple mechanisms may contribute to transitive inference as a function of the specific experimental setting under consideration (Zeithamova et al. 2012), it should be noted that a contextual transfer effect favors the operation of “encoding-based” models in this experimental setting (e.g., the temporal context model [TCM)] [Howard et al. 2005; Polyn and Kahana 2008]), over “retrieval-based” models (Kumaran and McClelland 2012) (e.g., see Zeithamova et al. (2012) for overview of both classes of models). Though TCM was originally developed to account for behavioral data in tasks involving free recall (Polyn and Kahana 2008), it has more recently been proposed to account for transitivity behavior in the TI paradigm (Howard et al. 2005). Briefly, TCM argues that the contextual representation of each item in a training pair (e.g., A and B) comes to share common features due to their temporal co-occurrence. On the assumption that rank information can be considered part of an item’s contextual representation, TCM might naturally produce the kind of contextual transfer effect observed—though it would be potentially illuminating for quantitative simulation studies to explore this issue in greater detail.

In summary, the current study developed a paradigm to characterize the effects of prior knowledge in facilitating new learning using a well-established experimental scenario, the transitive inference task. Our findings demonstrate that an associate schema has a striking effect on the learning of a new hierarchy, even in surprising conditions (i.e., when corrective feedback is entirely absent), and provides initial insights into the underlying cognitive mechanisms of such schema-driven effects. In the future, it will be interesting to further characterize the underlying cognitive and neural mechanisms using quantitative modeling studies and functional neuroimaging, respectively.

## Materials and Methods

### Stimuli

Pictures of galaxies were obtained from various sites on the internet (including http://hubblesite.org/gallery/album/nebula) (Fig. 1). The allocation of individual pictures to position in the hierarchy was randomized across the group of participants. Stimuli were presented using Cogent Graphics toolbox (http://www.vislab.ucl.ac.uk/cogent_graphics.php) operating in a Matlab 7 environment.

### Participants

Thirty healthy individuals participated in this experiment (age range 19–33, 16 female). All participants gave informed written consent to participation in accordance with the local research ethics committee.

Each group (Groups I and II) comprised 15 participants. There were no significant differences between subject groups in terms of age or years of higher education (*P*’s > 0.1). Note that Groups I and II were treated identically in phase 1, and only differed with regards to the order of hierarchy presentation (i.e., whether the first train–test cycle was FAMILIAR [Group I] or NEW [Group II]) (see below). The critical difference between groups was during phase 3: Participants in Group I were exposed to a hierarchy which contained items derived from the phase 2 NEW hierarchy (i.e., a “weak schema” condition), whereas those in Group II were exposed to a hierarchy containing items derived from phase 2 FAMILIAR hierarchy (i.e., “strong schema” condition).

### Experimental design

There were three experimental phases (i.e., 1–3) (Fig. 1), all involving the TI paradigm—these were divided into five sessions (two sessions each for phase 1 and 2, and one session for phase 3) separated by a break of 1–2 min each.

In phase 1, all participants completed 20 training and 20 test blocks of the TI paradigm, involving a new seven-item galaxy hierarchy (i.e., A–B–C–D–E–F–G, where A is the highest ranking item and G the lowest). Each training block comprised six training trials (e.g., A vs. B, see below) and six test trials (e.g., B vs. D, see below), with order of training and test trial presentation being pseudorandom.

In phase 2, participants completed 12 training and 12 testing blocks involving two nine-item galaxy hierarchies (i.e., A–B–C–D–E–F–G–H–I). Prior to the start of phase 2, participants received instructions as follows: “You will see two sets of galaxies. One set of galaxies will be entirely new. The other set of galaxies will contain galaxies you have previously learned about, as well as new ones. What you learned about galaxies you have learned about before will still hold true, but you need to also learn about the new galaxies.”

Critically, therefore, five of the items in the “FAMILIAR” hierarchy (i.e., A, C, E, G, I) had been part of the hierarchy learned during phase 1 (i.e., the B, C, D, E, F items, respectively) (Fig. 1). The other four items (i.e., B, D, F, H) in the “FAMILIAR” hierarchy were new items that had not been seen before. In contrast, all nine items in the “NEW” hierarchy (i.e., items A–I) were novel items that had not been previously experienced. During phase 2, participants completed alternating train–test cycles of each of the two hierarchies. Participants in Group I viewed the FAMILIAR hierarchy in the first train–test cycle, and those in Group II viewed the NEW hierarchy in the first cycle (to control for potential order effects). The accuracy with which participants could reconstruct the hierarchies (the hierarchy recall test, see below) was tested after phases 2 and 3.

In phase 3, participants completed six training and testing blocks involving one nine-item galaxy hierarchy (i.e., A–B–C–D–E–F–G–H–I). Prior to the start of phase 3, participants were told that they would be exposed to a new set of galaxies, and that: (1) previously acquired information about the oldness of galaxies would still hold true; (2) there would be no feedback of any kind (i.e., no corrective or cumulative feedback); and (3) the way in which galaxies are presented (e.g., during training trials) would be the structured as in previous phases.

Participants were instructed as follows:

“In this phase of the experiment, you will once again have to learn about one new set of galaxies—some galaxies will be ones you haven’t seen before, and some will be familiar to you from phase 2.
“The task setup will be similar to before but there are a few key differences to bear in mind: As before, what you learned before about which galaxies are older is still true; for example if galaxy X was older than galaxy Y, then this will still be the case in this phase. So you’ll be able to use knowledge gained from phase 2 to help you here. There will also be training trials and test trials. However, you won’t be given feedback during training trials in this phase. What this means is that you, of course, won’t be able to learn about which galaxies are older from the feedback—we can’t tell you exactly how to learn which galaxies are older but we can tell you that it is still possible to do pretty well, and we can give you a few hints:
“You should pay close attention to the way in which galaxies are presented, i.e., which galaxies are appear together as pairs. We can also tell you that the way in which galaxies are paired together will remain the same as in previous phases of the experiment. So even though you may find this phase difficult, just do your best, and you should try to use this information to guide your performance, both during training trials and also during test trials. If you’re unsure, go with your gut instinct. Note that we’ll still score your responses and performance during training and test trials will count toward final payout. The third difference from before is you also won’t be shown cumulative totals and the end of each block, but as mentioned before, we’ll tell you at end how you did.”

The critical manipulation was that hierarchy composition varied according to participant group: In Group I, five of the items (i.e., A, C, E, G, I) in the phase 3 hierarchy were derived from the NEW hierarchy in phase 2 (i.e., the B, C, D, E, F items, respectively). In Group II, five of the items (i.e., A, C, E, G, I) in the phase 3 hierarchy were derived from the FAMILIAR hierarchy in phase 2 (i.e., the A, C, E, G, I items, respectively). For both groups, the other four items (i.e., B, D, F, H) were novel and had not been previously experienced. In this phase, no feedback was presented on training (or test) trials—nor was cumulative feedback at the end of each block provided. The set-up of phase 3, therefore, allowed us to examine whether the presence of a prior schema is sufficient to support successful transitivity performance even in the absence of corrective feedback. Further, we assessed the effect on performance of manipulating the strength of the associative schema; as described above, in Group II, previously seen items in the phase 3 hierarchy were both more familiar (i.e., had been seen during phase 1) and occupied exactly the same positions in the hierarchy as in the previous phase (i.e., phase 2) (Fig. 1).

In all phases (including phase 3), the start of each block was preceded with the relevant instruction (i.e., “Get ready for training trials,” “Get ready for test trials”). Following the end of each block in phases 1 and 2, a screen showing the percentage of correct responses achieved was displayed.

### Training and test trials

During a training trial, adjacent items in the hierarchy were presented on either side of the screen (i.e., phase 1, six premise pairs A vs. B, B vs. C, C vs. D, D vs. E, E vs. F, F vs. G; phases 2 and 3, eight premise pairs, i.e., A vs. B, B vs. C, C vs. D, D vs. E, E vs. F, F vs. G, G vs. H, H vs. I). The left–right position of an item on the screen was randomized across trials. Participants had up to 3 sec in which to choose, via button press (i.e., left or right, index or middle finger of the right hand, respectively), the item which they thought was “older.” In phases 1 and 2 (but importantly *not* phase 3), corrective feedback was issued after the participant’s choice had been made. This consisted of a green square border which indicated the participant’s choice together with either “+20 points” or “−20 points” for a correct or incorrect response, respectively. A fixation cross preceded the onset of the next trial.

Note that our use of a relation that is transitive in nature (i.e., “older”) follows the original version of the TI paradigm developed by Bryant and Trabasso (1971). In other work, we show that the use of a well-specified transitive relation during the instructions, as opposed to one that is ambiguous in meaning and typically used in TI experiments (i.e., “correct”), increases the success of transitivity performance and ensures the development of robust knowledge of the hierarchy across a subject group (Kumaran et al. 2012).

Test trials involved the presentation of nonadjacent items in the hierarchy. As in training trials, participants had 3 sec in which to choose, via button press (i.e., left or right), the galaxy which they thought was “older.” The composition of test trials differed across phases: In phase 1, the following six test trial types were presented: B–D, B–E, B–F, C–E, C–F, D–F. In phases 2 and 3, the six test trial types were: B–D, B–F, B–H, D–F, D–H, F–H. Critically, therefore, items presented during test trials in phases 2 and 3 had not been experienced in a previous phase (i.e., were novel items in that phase).

Importantly, feedback was not presented during test trials, though participants were instructed that their choices would still count toward their final payout. Instead, a screen appeared which required participants to rate (on a scale of 1 to 3) their confidence in their decision: Participants were carefully instructed to enter a “1” response if they were guessing entirely, a “2” response if they “had some idea but were not sure” about their choice, and to reserve a “3” response if they were “>90% certain” that their choice was the correct one. Participants were told that though their confidence responses would not count toward their final payout, they should still answer as accurately as possible. Note that although participants’ confidence responses were obtained in this experiment, here we restrict our analyses to the binary choice data.

The remuneration received by participants was determined directly from the number of correct responses during training and test trials, in addition to a basic minimum for participation in the experiment.

### Hierarchy recall test

A hierarchy recall test in which participants were asked to explicitly reconstruct the order of items in the hierarchy was used (Smith and Squire 2005; Kumaran et al. 2012). Subjects were shown pictures of the set of galaxies, displayed in a random arrangement on a tabletop, and asked to reconstruct the correct order of galaxies in the hierarchy. Participants’ performance on the hierarchy recall test was scored using a procedure which penalizes incorrect positioning of an item according to its deviation from the item’s true position. Specifically, the summed deviation of a participant’s stated position of each item from its true position (e.g., if the top ranked item was placed in 6th position, this would be scored as a deviation of five) was calculated.

### Behavioral analyses

These were conducted using SPSS version 19 using standard procedures. The analyses presented here focus on the binary choice data obtained during training and test trials; the test trial confidence data are not considered for the present purposes. Performance accuracy and reaction time were analyzed for both training blocks and test blocks, using repeated measures mixed factor analyses of variance (ANOVA). Mauchly’s test was used to evaluate whether the assumption of sphericity had been violated, and degrees of freedom were corrected using Greenhouse–Geisser estimates of sphericity when appropriate.
